# Activity of antimicrobial examination gloves under realistic conditions: challenge not fulfilled

**DOI:** 10.1186/s13756-023-01322-z

**Published:** 2023-10-24

**Authors:** Eva-Maria Klupp, Birte Knobling, Gefion Franke, Cristina Belmar Campos, Philipp M. Maurer, Johannes K. Knobloch

**Affiliations:** Department for Infection Prevention and Control, Institute for Microbiology, Virology and Hygiene, University Medical Center Hamburg-Eppendorf, Martinistraße 52, 20251 Hamburg, Germany

**Keywords:** Antimicrobial surfaces, Examination glove, Light-activated antimicrobial properties, Gram-positive microorganisms

## Abstract

**Background:**

Antimicrobial materials or surfaces are advertised as part of infection prevention bundles. However, the efficacy of such antimicrobial surfaces has not been sufficiently investigated in hospitals. In this study, the antimicrobial activity of examination gloves with light-activated antimicrobial properties against Gram-positive microorganisms was investigated modelling real live conditions.

**Method:**

In a standardized experimental set-up with dry and realistic contamination, the antimicrobial properties of gloves claiming light dependent antimicrobial activity against Gram-positive organisms were tested in comparison with conventional examination gloves. All gloves were contaminated through a standardized activity of the test persons for construction with contaminated building blocks. For contamination suspensions of *Enterococcus faecium* ATCC 6057, *Acinetobacter baumannii* (outbreak strain), methicillin resistant *Staphylococcus aureus* ATCC 43300 or *E. faecium* (VRE) patient isolate were dried on the surfaces. After the standardized activity, the gloves were held for 10 min in the light present in the room (bright conditions) and the grade of contamination was determined subsequently by quantitative culture. In one experimental series gloves were held in a dark box after contamination as a control (dark conditions).

**Results:**

The light intensity in all experiments under bright conditions was significantly above the limit value specified by the manufacturer for the activation of antimicrobial properties (> 500 lx). The mean values for experiments with antimicrobial active and non-active gloves were 955 and 935 lx, respectively. As claimed by the manufacture, the gloves showed no sufficient efficacy against *A. baumannii* under bright conditions. Against Gram-positive microorganisms such as *E. faecium*, *E. faecium* (VRE) and methicillin resistant *S. aureus* the gloves showed only very low antimicrobial activity with a reduction factor < 1 log_10_ even after 10 min in bright conditions. Interestingly, comparable results for experiments *with A. baumannii* and *E. faecium* were shown under dark conditions.

**Conclusion:**

The lack of activity of the active principle against Gram-negative microorganisms could be confirmed. The reduction factors of > 4 log_10_ within 5 min for Gram-positive microorganisms claimed for the product using a standard test procedure (ASTM D7907) could not be confirmed in a realistic experimental test set-up even after 10 min of light exposure. The effectiveness against Gram-positive microorganisms should be further investigated under realistic (dry) conditions, including patient care. At this stage, the use of supposedly antimicrobial gloves should not be recommended, as the belief in their efficacy may encourage the misuse of gloves.

**Supplementary Information:**

The online version contains supplementary material available at 10.1186/s13756-023-01322-z.

## Background

Nosocomial infections are a well-known challenge in patient care. They lead to high mortality and morbidity rates and also to high overall costs for hospitals and massive financial losses for the health care system [1, 2]. An estimated 90% of exogenously caused nosocomial infections are transmitted via the hands of medical personnel [3]. For this reason, hand hygiene including the correct use of medical gloves plays a central role in the prevention of nosocomial infections. Staff compliance with hand antisepsis has a direct impact on the transmission of potentially pathogenic agents and the development of nosocomial infections [4–6]. Apart from hand hygiene, wearing gloves also has an important role in the prevention of hospital acquired infections [4, 6, 7]. Despite knowledge of hand antisepsis and proper glove use as important components of nosocomial infection prevention, they are not fully followed and compliance rates are sometimes low depending on the indication [4, 8, 9].

During patient care, gloves can become heavily contaminated. Depending on the type of patient care and the duration of usage, the degree of contamination of medical gloves can range from 2 colony forming units (cfu) to more than 3 × 10^4^ cfu per glove [9, 10]. Others found rates from 5 cfu to more than 300 cfu when examining only gloved fingertips after patient care [11]. Incorrect glove use can therefore facilitate the spread of microorganisms and can affect cross-contamination among patients [5, 9, 12, 13].

Antimicrobial materials and antimicrobial surfaces are therefore increasingly coming into focus and are being promoted for many hygiene-relevant areas as a measure for infection prophylaxis. As an innovation, antimicrobial gloves with different active principles, for example with Polyhexamethylenbiguanid (PHMB) [10], with integrated antiseptic dyes [14], ClO_2_ generating gloves [15] or gloves releasing reactive oxygen species (ROS) [16] have been launched on the market in recent years. However, the effectiveness of such antimicrobial surfaces and gloves in the reality of patient care has not been sufficiently investigated [17].

According to the manufacturer instructions for gloves with light activated antimicrobial activity [16] a 4 to 5 log_10_ reduction within 5 min could be observed in Gram-positive bacteria, including multi-resistant strains such as MRSA and VRE. For Gram-negative bacteria, the reduction is reported to be between 1 and 1.5 log_10_ after 10 min with this product. The active principle of these gloves is based on a dye that is firmly integrated into the outer surface of the glove and is designed to catalyze the formation of singlet oxygen when exposed to light and oxygen. Singlet oxygen acts as an oxygen radical (ROS) and is said to attack the cell membrane of (mainly Gram-positive) bacteria, ultimately leading to cell death [16, 18–21].

The testing of the antimicrobial effect of gloves to claim antimicrobial activity is carried out by means of testing according to ASTM D7907 [22]. Comparable to the ISO 22196 [23] for non-porous surfaces the ASTM D7907 test principle is based on transferring a suspension containing the bacterial strain to be tested to a 10 cm^2^ test sample of the antimicrobial glove and quantitatively determining the amount of bacteria after various defined contact times of the liquid. The relative reduction is subsequently calculated of the log_10_ count of the test surface compared with the log_10_ value of a non-antimicrobial control.

However, this test principle does not reflect the real conditions in patient care. When handling patients, gloves are not usually exposed to a large amount of contaminated liquid for several minutes, as glove change is suggested in the case of visible contamination. Therefore, for infection-preventive efficacy in patient care, rapid efficacy against unvisible dry contamination with microorganisms would also have to occur, which cannot be assessed after testing with ASTM 7907. The lack of validity of liquid-based test methods like the ISO 22196 for antimicrobial activity against dry soiling has already been demonstrated for non-porous solid surfaces [24, 25]. Therefore, statements on other surfaces evaluated as antimicrobial by means of liquid-based test methods should also be critically questioned. Therefore, we developed an alternative test for antimicrobial-equipped (ae) gloves simulating realistic conditions in patient care resulting in dry contamination of the gloves with clinically relevant organisms. Using this test, the antimicrobial activity of antimicrobial examination gloves with ASTM D7907 confirmed activity against Gram-positive bacteria were analysed.

## Methods

The antimicrobial properties of light activated antimicrobial gloves (B. Braun, Germany; batch number 1809375181) were investigated in a standardized test set-up in comparison with conventional test gloves from the same manufacturer (VASCO® Nitril Soft white; B. Braun, Germany).

For the purpose of contamination, *A. baumannii complex* (bla_OXA-23_ positive clinical outbreak isolate), *E. faecium* ATCC 6057, vancomycin resistant *E. faecium* (VRE, clinical isolate, VanB, sequence type ST117) or methicillin resistant *S. aureus* ATCC 43300 (MRSA) were used. Overnight cultures of the respective isolates were used to prepare a bacterial suspension of McFarland 4.0 in 2 mL of low organic load (0.3 g/L bovine albumin serum (BSA); BIOMOL GmbH, Germany) to achieve a sufficient and representative bacterial load for the situation of non-visual contamination of the hand contact surface.

For contamination of the hand contact surface (play brick wall), 1500 µL of the bacterial suspension was transferred onto a sterile gauze compress. The wetted gauze was wiped over all available sites (total surface: 76,592 cm^2^) of a disinfected wall of play bricks (Additional file 1: Fig. S1A) and the wall was dried to generate primary contaminated surfaces (PCS). The play brick wall consisted of bricks of different sizes in two different colors, which were built in alternating colors.

According to a standardized building instruction, all subjects (n = 10) used gloved hands to disassemble the wall into its individual parts and subsequently build a tower from the alternately colored play brick wall, first using the bricks of one color and then those of the other color (Additional file 1: Fig. S1B–E). This defined and standardized work order ensured that all subjects had a similar probability of contaminating their gloves. Following this standardized activity, the gloves were held with the palm open and facing upwards for 10 min in the light present in the room (bright conditions) to activate the dye integrated into the glove and form ROS. The intensity of the light was determined by luxmeter [EBLX-3; Hartmann & Braun (Additional file 1: Fig. S1F)].

The degree of contamination of the gloves was subsequently determined by quantitative culture. To recover the bacteria, the gloved hands were immersed in a Stomacher bag (Hassa GmbH, Germany) containing 400 mL of NaCl for 30 s, separately on the left and right sides, while making kneading and wiping hand movements (Additional file 1: Fig. S1G). 50 mL of the NaCl solution of each Stomacher bag was applied by membrane filtration on 0.2 μm nitrocellulose membrane (Merck Millipore, Germany) in duplicate to McConkey Agar (*A. baumanni complex;* Biomérieux, France), Slanetz and Bartley Agar (*E. faecium;* ThermoFisher, USA) or Chapman Agar (*S. aureus;* ThermoFisher, USA) depending on the bacterial strain used, and incubated at 37 °C for 24 h (*A. baumanni complex*) or 48 h (*E. faecium*, *S. aureus*)*.* The colonies grown on the plates were counted (colony forming units, cfu) and the mean value of both approaches was determined. Each subject performed the experimental set-up with antimicrobial as well as non-antimicrobial control gloves, although the subjects were unaware of which glove was being worn. In addition, the subdivision of the handedness of the sample ends into dominant and non-dominant hand was performed to investigate/exclude a possible influence of the handedness on the experiment.

In order to investigate the influence of light on the activation of the dye incorporated in the gloves, additional experiments with analoge set-up were performed under dark conditions. Therefore, the hand contact surface of (n = 10) subjects has been contaminated with *E. faecium* ATCC 6057 and A*. baumannii complex* by the standardized activity. However, the subsequent 10-min waiting period was waited in a box, generating dark conditions. The light intensity was also controlled by luxmeter during this process. The experiments with MRSA and VRE were performed only under bright conditions.

To calculate the log_10_ reduction values (LRV), log_10_ count of the antimicrobial gloves was subtracted from the log_10_ count of control gloves in each experiment. The median or mean value was then calculated from the LRVs from all experiments.

To assess differences in the degree of contamination between antimicrobial and non-antimicrobial control gloves for dominant and non-dominant hand, statistical analysis was conducted using R (version 4.2.2 [26]) and R studio (version 2023.03.1 [27]) with activated package *rstatix* [28]. One-way ANOVA was performed using the command *anova_test*. In the case the *p*-value was < 0.05, pairwise *t*-test (command *pairwise_t_test*) with bonferroni adjustment was carried out.

## Results

**Fig. 1:**
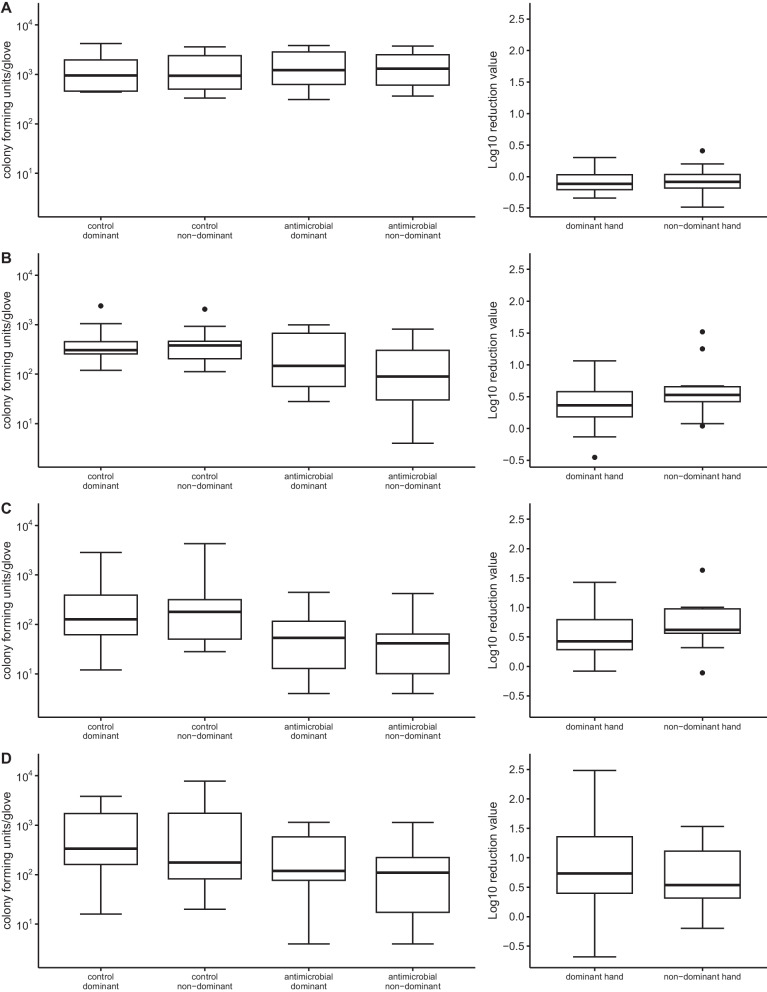
**A**–**D** Number of colony forming units per glove separated into dominant and non-dominant hand and comparison of antimicrobial effective glove (ae) versus control (left side) as well as log_10_ reduction values (right side) under bright conditions for four different strains tested **A** *A. baumannii* complex clinical outbreak strain **B** *E. faecium* ATCC 6057 **C** VRE clinical strain **D** MRSA ATCC 43300)

**Fig. 2:**
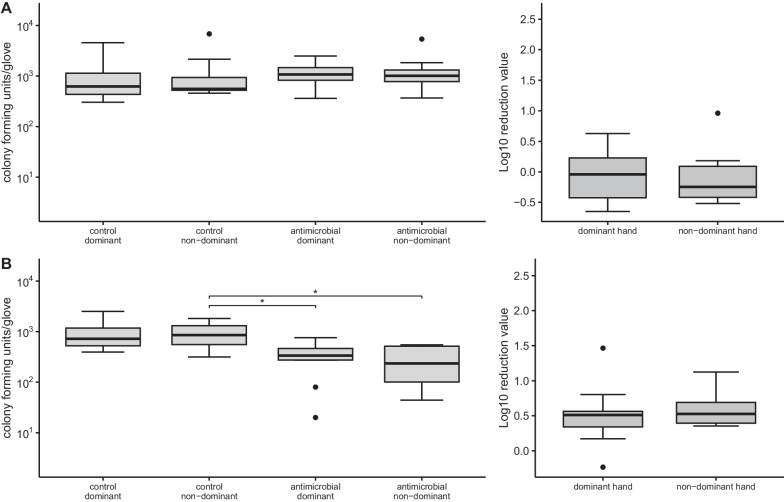
**A**, **B** Number of colony forming units per glove separated into dominant and non-dominant hand and comparison of antimicrobial effective glove (ae) versus control (left side) as well as log10 reduction values (right side) under dark conditions (**A** *A. baumannii complex* clinical outbreak strain **B ***E. faecium* ATCC 6057). Significant differences of the pairwise t-test are indicated by brackets and are marked with * (*p* < 0.05)

For all investigated isolates and volunteers well quantifiable numbers of bacteria were transferred from the PCS to the control gloves during the standardized activity even after the 10-min waiting period (Figs. 1 and 2). On the dominant hands of volunteers mean values of 1457.2, 574.8, 510.4, and 1036.8 cfu/glove as well as median values of 954, 306, 148, and 336 cfu/glove were observed for *A. baumannii*, *E faecium* ATCC 6057, VRE, and MRSA ATCC 43300, respectively. The amount of detectable bacteria displayed no significant differences with respect to the handedness of the individual subjects for none of the bacterial strains. Therefore, the standardized activity was confirmed to reach a suitable grade of contamination to investigate antimicrobial activity in the context of dry contamination.

During the experiments to investigate the efficiacy of light activated antimicrobial activity, light intensity in each experiment was significantly above the limit specified by the manufacturer for the activation of antimicrobial properties (> 500 lx, Additional file 2: Fig. S2). The mean values for experimental series ranged from 952.5 to 1407 lx (median 982.5–1462.5 lx).

Following exposure to light, the antimicrobial-equipped (ae) gloves showed no difference in residual microbial contamination for the Gram-negative strain of the *A. baumanii complex* for either the dominant or non-dominant hand of the subjects (Fig. 1A, Additional file 3: Table S1). On the ae gloves slightly more bacteria were observed (mean 1693.6 and 1624.4 cfu for dominant and non-dominant hands) compared with control gloves (mean 1457.2 and 1503.6 cfu) resulting in a mean LRV of −0.08 and −0.05 log_10_ (median −0.12 and −0.08 log_10_) for the dominant and non-dominant hand, respectively. One-way ANOVA showed no significant differences (F(3, 36) = [0.073], *p* = 0.974).

For Gram-positive organisms, a minor reduction of bacterial burden at the end of experiments was observed in comparison between ae and control gloves, none of which reached a statistically significant level (Fig. 1B–D, Additional file 3: Table S1). As shown for the control gloves, no influence of the handedness of the subjects on the measured microbial burden could be observed for the antimicrobial gloves either. For *E. faecium* ATCC 6057 a mean of 353.2 and 574.8 cfu for the dominant hand as well as 242 and 536.6 cfu for the non-dominant hand was observed for ae and control gloves, respectively. Thus, the relative reduction averaged 0.34 log_10_ for the dominant hand and 0.61 log_10_ (median 0.36 log_10_ and 0.53 log_10_) for the non-dominant hand (Fig. 1B). The clinical VRE strain displayed a similar pattern with a mean of 120.8 and 510.4 cfu for the dominant hand as well as 95.2 and 654.4 cfu for the non-dominant hand ae and control gloves, resulting in a mean LRV of 0.55 and 0.71 log_10_ (median 0.43 log_10_ and 0.62 log_10_), respectively (Fig. 1C). For MRSA ATCC 43300 a mean LRV of 0.90 and 0.66 log_10_ (median 0.73 log_10_ and 0.54 log_10_) was observed for the dominant and non-dominant hand with a mean of 288.2 and 1036.8 cfu for the dominant hand as well as 234.4 and 1584.8 cfu, respectively (Fig. 1D). For both organisms no significant differences could be observed (*E. faecium*: F(3, 36) = [0.927], *p* = 0.437; VRE: F(3, 36) = [1.228], *p* = 0.314; MRSA ATCC 43300: F(3,36) = [1.925], *p* = 0.143).

To investigate whether the observed slight reduction was caused by light-activatable antimicrobial activity, experiments were performed with *A. baumanii* complex and *E. faecium* ATCC 6057 under dark conditions considerably below the minimum amount of light for activation specified by the manufacturer (Fig. 2, Additional file 3: Table S1). Under these conditions for the *A. baumanii* complex isolate slightly more bacteria were observed on the ae gloves (mean 1168 and 1425.2 cfu for dominant and non-dominant hands) compared with control gloves (mean 1420.4 and 1380.8 cfu) resulting in a mean LRV of −0.07 and −0.10 log_10_ (median −0.04 and −0.25 log_10_) observed for the dominant and non-dominant hand, respectively (Fig. 2A; Additional file 3: Table S1). Interestingly, for *E. faecium* ATCC 6057 a reduction was observed under conditions lacking sufficient light with a mean of 368.4 and 999.2 cfu for the dominant hand as well as 292.4 and 965.6 cfu for the non-dominant hand for ae and control gloves, respectively. One-way ANOVA showed significant differences between groups resulting in F(3, 36) = 6.718, *p* = 0.001. The subsequent pairwise *t*-test showed that the differences between the ae glove dominant hand and the non-dominant control gloves (−595, 95% CI (−999, −195)) as well as ae glove non-dominant and control glove non-dominant hand (−673, 95% CI (−1071, −275)) were statistically significant (*p* = 0.042 and *p* = 0.019). Despite darkness a relative reduction averaged 0.50 log_10_ (median 0.51 log_10_) for the dominant hand and 0.60 log_10_ (median 0.53 log_10_) for the non-dominant hand was observed (Fig. 2B, Additional file 3: Table S1).

## Discussion

Antimicrobial surfaces and materials are frequently discussed as part of infection prevention bundles to reduce nosocomial infections. However, there is no evidence in the literature that antimicrobial gloves can prevent nosocomial infections. Due to the fact of frequent pathogen transmission occurring by the hands of medical staff [5, 9–11, 29] several manufacturers promote antimicrobial gloves based on different principles of action. Usually the potential antibacterial mode of action is confirmed by in vitro microbiological testing in accordance with ASTM D7907, a liquid based in vitro test that uses comfortable humidity and temperature, a large amount of contaminated liquid and unreallisticially long waiting periods.

In the recent years, it was demonstrated for solid surfaces that antimicrobial activity postulated by means of liquid-based in vitro testing is not representative for dry contamination typically observed on surfaces in hospitals [24, 25]. Therefore, results from liquid-based in vitro testing on flexible surfaces such as examination gloves should be critically reviewed. In patient care, gloves become invisibly contaminated by contact to patients and dry surfaces in the immediate environment. After contact with secretions and excretions of patients with visible contamination, an immediate change of gloves is recommended [6].

Therefore, the use of antimicrobial gloves in patient care would only make sense for longer periods of wear during activities without visible contamination with liquids and if the contamination of the gloves with microorganisms could be reduced immediately (hence in a contact time 3–5-s) [30]. Therefore, in the real world of patient care, gloves will thus never be exposed to the experimental conditions used in ASTM D7907. This finding is also supported by a current report of the VDI (The Association of German Engineers), which also confirms that it is not yet possible with normative methods to evaluate the contribution of antimicrobial products to the interruption of infection chains because their test design does not take into account practical application [17]. Therefore, in this study an alternative test method was established and gloves with antimicrobial activity detected in the ASTM D7907 test were examined.

The dry contamination with low organic load achieved in our experimental set up corresponds to realistic non visible contamination in patient care, which is why our test is basically suitable. In other studies, bacterial contamination of gloves after patient care ranged from 2 cfu/glove to > 30,000 cfu/glove or from 0 to more than 300 cfu per 5 fingertips, depending on the type and duration of patient contact and care, respectively [9–11]. These values are comparable to our range of 500–1500 CFU/glove on average measured in control gloves. The postulated antimicrobial gloves showed no activity at all for the Gram-negative species *A. baumannii complex*. However according to the manufacturer a reduction was also observed for Gram-negative bacteria using ASTM D7907 testing after 10 min. For the three different Gram-positive isolates tested, a marginal, not statistically significant reduction was found after 10 min waiting time with sufficient light intensity and was much lower compared to the reduction claimed by ASTM testing.

Because the activity is based on light according to the manufacturer's information, control experiments were carried out without light. The product's postulated antimicrobial activity is caused by a dye integrated into the outer layer of the gloves, which is said to act as a catalyst and generate the release of singlet oxygen in the presence of light. Singlet oxygen acts as a radical and attacks bacterial proteins and lipids, ultimately leading to cell death. Interestingly, the experiments without light showed a reduction of Gram-positive *E. faecium* after 10 min that was comparable to the LRV we have already seen with light. In this case observed differences in mean reduction were statistically significant (*p* < 0.05)*.* However, the confidence interval is quite large and statistical significance can be assessed as insufficiently meaningful in the light of the professional background. Furthermore, the reduction was observed under dark conditions, so it may not be attributed to the claimed mode of action of the gloves, which is light-dependent. Instead, it could be assumed that the statistical significance is among others due to naturally occurring variations in the growth behaviour of microorganisms.

Also it was already shown that the glove material is an important factor influencing the bacterial transfer to and from a gloved hand [8]. Maybe the slight differences we observed in this study was due to a lower adherence of the bacteria to the glove material with the integrated dye instead of an active reduction.

Our study has some limitations which might be addressed in future research. Since only a specific batch of the manufacturer's antimicrobial gloves was studied, the statements in this study refer only to this batch. No general statement can be made at this time. This work also does not allow any general statements to be made about other gloves with postulated antimicrobial efficacy whose active principle is based on a different method.

In addition to the questionable effectiveness in practice, further aspects have to be considered when assessing the meaningfulness of such gloves. It should be mentioned that any disposable medical gloves must be removed after the end of an activity. Their change correlates with the indications for hand antisepsis [9]. This must always be done after the gloves have been removed, as all gloves do not provide complete protection against contamination of the hands through undetected perforations and also risk of contamination if the gloves are not removed properly. A deviation from this may be necessary in situations where frequent glove changes would have a relevant negative impact on the workflow. Here, disinfection of gloved hands would be conceivable. However, this would only make sense in the case of rapid, immediate effectiveness. A continuous reduction over a longer period of time by antimicrobial active ingredients cannot have a meaningful effect in this use. Moreover, the development of antimicrobial gloves with only (potential) efficacy against gram-positive pathogens should be discussed very critically, as the practical relevance is highly questionable. Furthermore, for reasons of sustainability, new gloves should only be developed if they are also biodegradable.

## Conclusion

In contrast to the standard test procedure (ASTM 7907) used by the manufacturer for claiming light-activated antimicrobial activity of gloves, our realistic experimental test set-up showed no antibacterial effect on Gram-negative or Gram-positive pathogens, even after 10 min of light exposure. When using this batch of gloves in patient care, no added value in terms of infection prevention is to be expected. Future-proof, optimized hygiene in healthcare therefore requires laboratory, field and benchmark tests that help to accurately evaluate the efficacy of antimicrobial agents. In general, the following applies to antimicrobial surfaces: They serve as a supplement to infection prevention and under no circumstances replace the applicable standard precautions. We are concerned that the advertising of supposedly “antimicrobial effective” gloves may give the user a false sense of security thus reduce compliance with correct glove changing and hand antisepsis and so leading to very risky health practices. As a result, the gloves may do the opposite of what was intended.

### Supplementary Information

**Additional file 1: Figure S1**: **A–G** Standardized experimental set-up with dry contamination. **A** Primary contaminated surface consisting of a play brick wall. **B–E** Standardized activity with building blocks for each test person: After putting on the (antibacterial) gloves the subjects were asked to build a tower from the alternating colored play brick wall, first using the bricks of one color and then those of the other color. **F** The gloves were held with the palm side open and facing upward for 10 min in the light present in the room to possibly activate the dye incorporated into the gloves to build ROS. Light intensity at the position of the hands was measured during all experiments.** G** The subjects dipped their gloved hands into a Stomacher bag containing 400 mL NaCl for 30 s, separately on the left and right side, while making kneading and wiping hand movements. The degree of contamination was determined by quantitative culture after membrane filtration.

**Additional file 2: Figure S2** Light intensitiy (median) measured in lx during all experiments to investigate the influence of light. Light intensity in each experiment with light was significantly above the limit specified by the manufacturer for the activation of antimicrobial properties (500 lx). Light intensity in each experiment in darkness was significantly under this limit.

**Additional file 3: Table S1** Results of the individual experiments.

## Data Availability

The datasets used and/or analysed during this study are included in this published article and its supplementary information files, further inquiries can be directed to the corresponding author.
