# Supplementary figures and images for: Activity of antimicrobial examination gloves under realistic conditions: challenge not fulfilled
Source: Antimicrob Resist Infect Control. 2023 Oct 24;12:116. doi: 10.1186/s13756-023-01322-z (PMC10599005; doi:10.1186/s13756-023-01322-z)

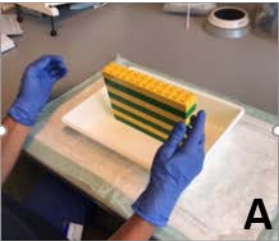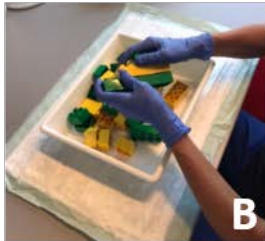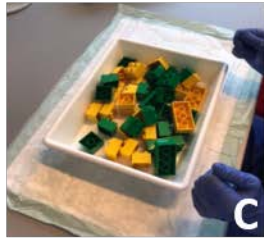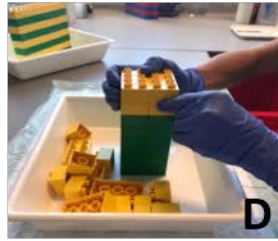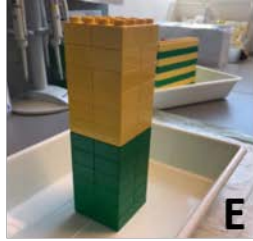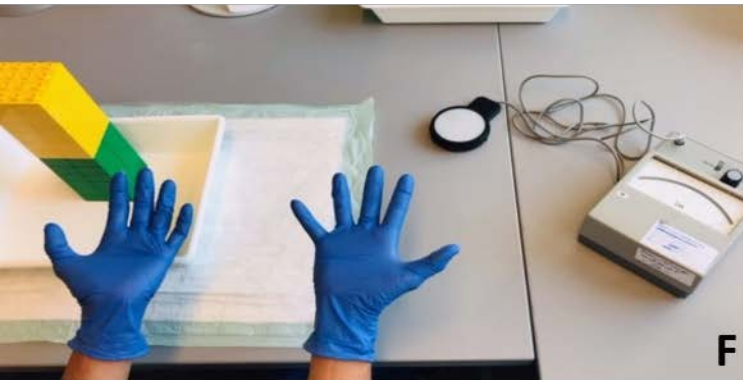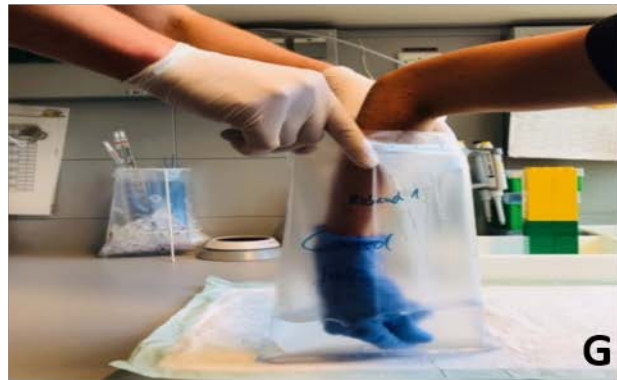

Supplement: Supplementary file 1 — Additional file 1: Figure S1: A–G Standardized experimental set-up with dry contamination. A Primary contaminated surface consisting of a play brick wall. B–E Standardized activity with building blocks for each test person: After putting on the (antibacterial) gloves the subjects were asked to build a tower from the alternating colored play brick wall, first using the bricks of one color and then those of the other color. F The gloves were held with the palm side open and facing upward for 10 min in the light present in the room to possibly activate the dye incorporated into the gloves to build ROS. Light intensity at the position of the hands was measured during all experiments. G The subjects dipped their gloved hands into a Stomacher bag containing 400 mL NaCl for 30 s, separately on the left and right side, while making kneading and wiping hand movements. The degree of contamination was determined by quantitative culture after membrane filtration. [file 13756_2023_1322_MOESM1_ESM.pdf]

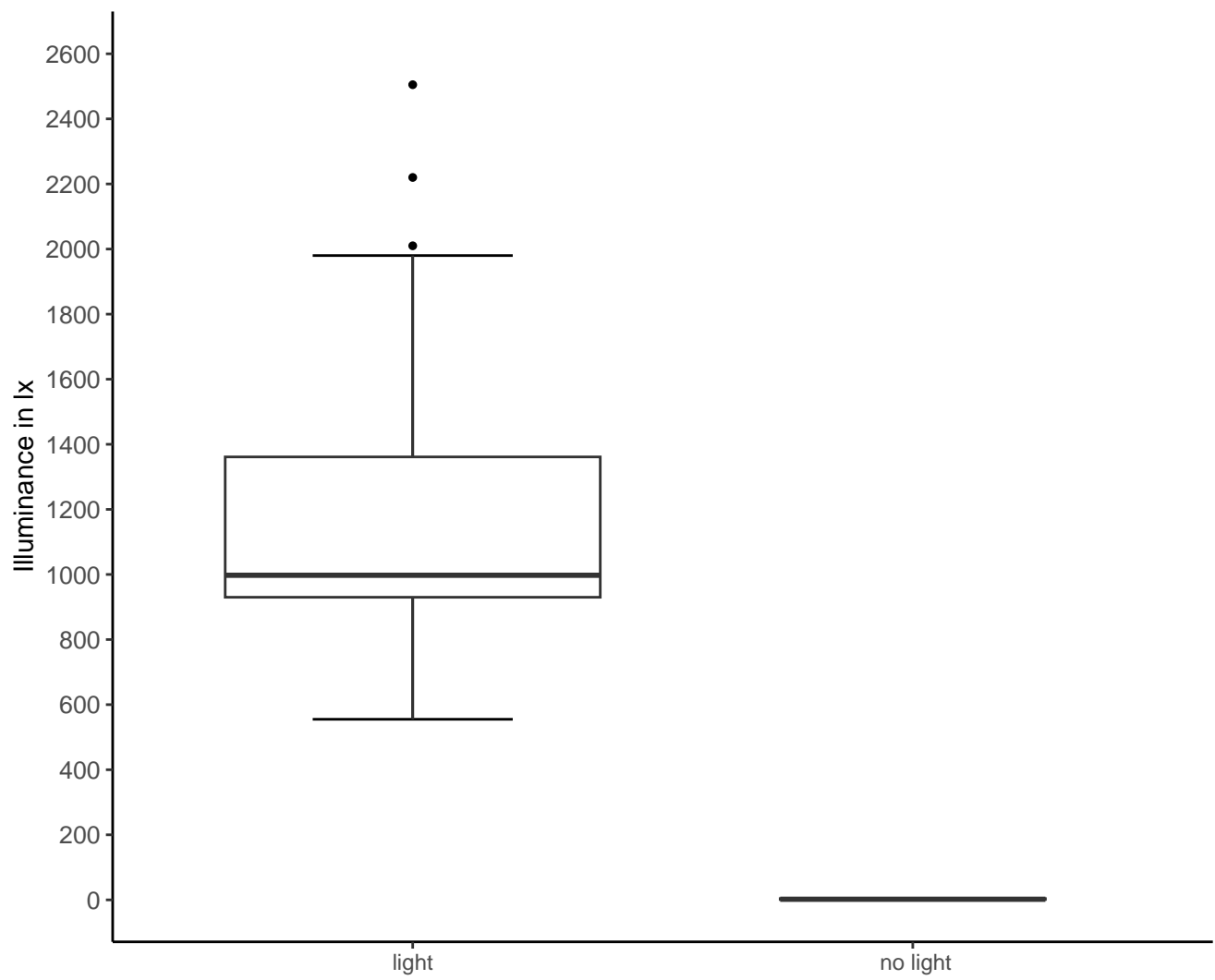

Supplement: Supplementary file 2 — Additional file 2: Figure S2 Light intensitiy (median) measured in lx during all experiments to investigate the influence of light. Light intensity in each experiment with light was significantly above the limit specified by the manufacturer for the activation of antimicrobial properties (500 lx). Light intensity in each experiment in darkness was significantly under this limit. [file 13756_2023_1322_MOESM2_ESM.pdf]
