# Supplementary material for: Activity of antimicrobial examination gloves under realistic conditions: challenge not fulfilled
Source: Antimicrob Resist Infect Control. 2023 Oct 24;12:116. doi: 10.1186/s13756-023-01322-z (PMC10599005; doi:10.1186/s13756-023-01322-z)
Supplement: Supplementary file 3 — Additional file 3: Table S1 Results of the individual experiments. [file 13756_2023_1322_MOESM3_ESM.docx]

**Supplementary table 1**

Results of the individual experiments

| Isolate | condition | hand | antimicrobial-equipped glove (cfu/glove) | | | | | | | | | | | | control glove (cfu/glove) | | | | | | | | | | | | p-value | mean LRV | median LRV |
| --- | --- | --- | --- | --- | --- | --- | --- | --- | --- | --- | --- | --- | --- | --- | --- | --- | --- | --- | --- | --- | --- | --- | --- | --- | --- | --- | --- | --- | --- |
|  |  |  | ex1 | ex2 | ex3 | ex4 | ex5 | ex6 | ex7 | ex8 | ex9 | ex10 | mean | median | ex1 | ex2 | ex3 | ex4 | ex5 | ex6 | ex7 | ex8 | ex9 | ex10 | mean | median |  |  |  |
| *A. baumannii* | light | dominant | 460 | 3144 | 3844 | 312 | 3192 | 952 | 596 | 732 | 1560 | 2144 | 1693.6 | 1256 | 928 | 3164 | 4212 | 440 | 2452 | 468 | 456 | 448 | 1024 | 980 | 1457.2 | 954 | 1 | -0.08 | -0.12 |
| *A. baumannii* | light | non-dominant | 364 | 3748 | 2932 | 472 | 2576 | 1228 | 592 | 660 | 2288 | 1384 | 1624.4 | 1306 | 936 | 3604 | 3200 | 332 | 2720 | 404 | 944 | 424 | 1624 | 848 | 1503.6 | 940 | 1 | -0.05 | -0.08 |
| *A. baumannii* | darkness | dominant | 804 | 1540 | 1072 | 1276 | 2484 | 360 | 1084 | 1652 | 888 | 520 | 1168 | 1078 | 404 | 376 | 1184 | 1016 | 556 | 704 | 4600 | 4520 | 304 | 540 | 1420.4 | 630 | 1 | -0.07 | -0.04 |
| *A. baumannii* | darkness | non-dominant | 860 | 1352 | 944 | 1084 | 1848 | 368 | 748 | 5400 | 1212 | 436 | 1425.2 | 1014 | 464 | 512 | 1060 | 644 | 560 | 560 | 6840 | 2156 | 456 | 556 | 1380.8 | 560 | 1 | -0.1 | -0.25 |
| *E. faecium* ATCC 6057 | light | dominant | 52 | 636 | 988 | 72 | 28 | 168 | 128 | 36 | 684 | 740 | 353.2 | 148 | 120 | 468 | 2400 | 316 | 172 | 256 | 296 | 416 | 1044 | 260 | 574.8 | 306 | 0.97 | 0.34 | 0.36 |
| *E. faecium* ATCC 6057 | light | non-dominant | 96 | 428 | 812 | 60 | 24 | 84 | 24 | 108 | 780 | 4 | 242 | 90 | 340 | 468 | 2060 | 192 | 112 | 248 | 428 | 448 | 928 | 132 | 535.6 | 384 | 0.88 | 0.61 | 0.53 |
| *E. faecium* ATCC 6057 | darkness | dominant | 360 | 680 | 280 | 20 | 764 | 80 | 476 | 436 | 272 | 316 | 368.4 | 338 | 1228 | 396 | 896 | 584 | 2504 | 508 | 1776 | 1060 | 572 | 468 | 999.2 | 740 | 0.09 | 0.5 | 0.51 |
| *E. faecium* ATCC 6057 | darkness | non-dominant | 548 | 256 | 84 | 44 | 460 | 60 | 544 | 536 | 176 | 216 | 292.4 | 236 | 1344 | 580 | 1120 | 396 | 1820 | 316 | 1248 | 1632 | 652 | 548 | 965.6 | 886 | 0.06 | 0.6 | 0.53 |
| *E. faecium* VRE | light | dominant | 16 | 128 | 12 | 396 | 448 | 84 | 4 | 8 | 40 | 72 | 120.8 | 56 | 428 | 296 | 68 | 1052 | 2860 | 224 | 32 | 12 | 72 | 60 | 510.4 | 148 | 0.70 | 0.55 | 0.43 |
| *E. faecium* VRE | light | non-dominant | 8 | 68 | 20 | 284 | 424 | 52 | 4 | 8 | 36 | 48 | 95.2 | 42 | 344 | 244 | 160 | 1100 | 4292 | 200 | 40 | 36 | 28 | 100 | 654.4 | 180 | 0.41 | 0.71 | 0.62 |
| *S. aureus* ATCC 43300 | light | dominant | 96 | 60 | 520 | 148 | 4 | 0 | 0 | 1152 | 816 | 84 | 288.2 | 90 | 1840 | 1452 | 3836 | 368 | 16 | 304 | 104 | 240 | 2068 | 140 | 1036.8 | 336 | 0.67 | 0.9 | 0.73 |
| *S. aureus* ATCC 43300 | light | non-dominant | 136 | 224 | 476 | 88 | 4 | 4 | 12 | 216 | 1132 | 52 | 234.4 | 112 | 4268 | 528 | 7828 | 56 | 20 | 136 | 76 | 228 | 2604 | 104 | 1584.8 | 182 | 0.19 | 0.66 | 0.54 |
